# Mining of Movie Box Office and Movie Review Topics Using Social Network Big Data

**DOI:** 10.3389/fpsyg.2022.903380

**Published:** 2022-05-26

**Authors:** Yinchang Chen, Zhe Dai

**Affiliations:** ^1^School of Media and Law, NingboTech University, Ningbo, China; ^2^Research Institute of Theatre Film and Television, Communication University of Zhejiang, Hangzhou, China

**Keywords:** movie box office, deep learning, big data, social network, box office prediction

## Abstract

In order to solve the problems of high investment and low box office losses in the film industry, this study analyzes the topic of film box office and film and television reviews based on social network big data. Firstly, the factors that affect the box office of the movie are analyzed. Secondly, continuous and discrete feature parts, text parts, and fusion parts are merged. The box office prediction model of mixed features using deep learning is established, and the movie box office is predicted. Finally, compared with other algorithms and models, the box office prediction model of mixed features using deep learning is verified. The results show that compared with other models, the prediction accuracy of the mixed feature movie box office prediction model using depthwise separable convolution (DSC)-Transformer is higher than that of other algorithm models. Its optimal mean square error (MSE) value is 0.6549, and the optimal mean absolute error (MAE) value is 0.1706. The constructed model predicts the box office of nine movies, and the error between the predicted value and the true value is about 10%. Therefore, the established movie box office prediction model has a good effect. This study can predict movies’ box office to reduce investment risk, so it is of great significance to movie investors and the social economy.

## Introduction

With the popularization of social networks, more and more users obtain various information through social network platforms, including news, entertainment, education, and orders. Meanwhile, users can also send comments and opinions through platform. Especially Weibo, as a very popular network platform, can be created and utilized. The timely extraction of valuable information from these data has become the research content of various industries (Palomba, 2020). Due to the development of social economy, people’s living standards are constantly improving. They are beginning to pay more attention to spiritual pursuits, and entertainment consumption is becoming more and more important in their lives. Movies account for a large part of entertainment consumption. Electrophoresis not only enriches people’s leisure life and spiritual world but also is an important means of cultural exchanges between countries (Galvão and Henriques, 2018; Ru et al., 2018).

At present, big data and cloud computing have become the most basic data forms and scientific computing methods in the current society. They not only save a lot of time and effort for users but also provide decision-making support for industries and enterprises. Big data are imported into the film industry from many aspects and plays an important role in film investment and financing, content creation, publicity and distribution, cinema terminals, and derivatives development. With the rapid development of the film industry, film production is also increasing. Movies are a high-risk industry, movie box office. It is not only affected by factors, such as movie plot, movie quality, screening time, movie scale, etc., but also by previous publicity, word of mouth, leading actors, and directors. Therefore, by predicting the box office of the movie, it is possible to adjust the initial investment, such as movie promotion, which can reduce the investment (Ahmed et al., 2020; Bogaert et al., 2021). Predicting the box office revenue of a movie before it is shown on the big screen has become an emerging demand. Forecasting movie box office can provide information for stock market investment decision-making, advertising company’s promotion strategy design, and movie theater broadcast density. This task is very challenging, and it is affected by many complex factors (Gaenssle et al., 2018; Franses, 2021; Kang, 2021). In the current era of big data, deep learning technology has a certain role in promoting massive data analysis. Wang et al. (2020) conducted a strategic investigation on the factors affecting the box office of movies and proposed a new framework composed of a series of feature learning models and prediction and ranking models. They used big data to model these factors to predict movie box office revenue. There are two specific learning feature models: (1) the new dynamic heterogeneous network embedding model can simultaneously learn the potential ideas of participants, directors, and companies; meanwhile, it can also capture their partnership and (2) the deep neural network-based model aims to reveal advanced representations of movie quality from the trailer. Using the learned features, train a mutually enhanced prediction and ranking model to obtain box office prediction results. Finally, the framework is applied to the Chinese film market. Comprehensive performance evaluation is performed using real data. The results show that the extracted knowledge and prediction results have good performance. There are many factors that affect the box office of a movie. The life cycle of a movie includes production, distribution, and exhibition factors. The most important costs are related to production factors. However, the title of a movie is one of the relatively inexpensive production factors that studios can use (Kim et al., 2018; Liu and Xie, 2019). Bae and Kim (2019) examined an informative movie title, that is, a movie title containing movie genre or storyline information. A 5-year analysis of the Korean film market shows that: information-rich movie titles have a positive impact on the box office revenue of under-advertised movies. Among them, the pre-release publicity activities are measured by the amount of media exposure before the release. From the perspective of selected features, the feature factors proposed by different scholars are also different. There are also different views on the influence of the same feature factors on the movie box office. This shows that the factors affecting movies’ box office and their mechanisms are very complex. Predicting movie box office is very difficult. When deep learning models are used to make predictions, they often only use simple BP neural networks and LSTM models, which cannot learn various features well.

One of the things that makes a movie good or bad is its box office. A high box office indicates that the movie is good, and investors will also consider the risks and benefits of the movie when investing in the movie. Therefore, if the model can accurately predict the box office of a movie, it can reduce the huge losses caused by investment risks to a certain extent and adjust reasonable shooting, production, publicity, and distribution strategies to maximize the return on investment. In order to solve this problem, a mixed feature movie box office prediction model based on deep learning is established to predict movie box office. It can reduce investment risk, which is significant to film investors and the social economy. The research idea is based on the problem that the encoding part of the existing Transformer model uses a fully connected feedforward neural network, which is computationally expensive and difficult to extract local invariant features. An improved depthwise separable convolution (DSC)-Transformer model is proposed. The depthwise separable convolution replaces the fully connected feedforward neural network in the encoding part and combined with the bidirectional encoder representation from transformers (BERT) model and the depthwise separable convolution, a hybrid feature prediction model is formed to predict and analyze the movie box office.

## Movie Box Office Prediction Algorithm and Model Design

### Analysis of Factors Affecting Movie Box Office

There are many factors that affect the box office of a movie. In movie box office prediction, some influencing factors are usually selected as variables. Not all factors can be used to predict the box office of a movie. For example, because the information is kept confidential, the producer will not disclose it online before the movie is released. There are also some factors such as search popularity, Internet word-of-mouth, and movie ratings that cannot be predicted. However, the characteristics of different characteristic data are different, so the data will also have different processing methods (Vujić and Zhang, 2018; Bao et al., 2021; Liao and Huang, 2021).

1. Director, actor, and screenwriter. The director plays an important role in the box office of a movie and determines the style, standard and quality of a movie. Actors are a key factor at the box office. An actor’s acting skills and influence will affect the box office. Screenwriting is also critical. This directly determines the direction and logic of the entire plot. If the plot of a movie is chaotic and unclear, it will directly affect the reputation of the movie. But these three factors cannot be directly used as variables to predict, so they need to be converted, as in Eq. (1):
(1) $$p_{i}=\frac{\overset{m}{\underset{j=1}{\sum }}b_{j}}{m}$$
  *p* is the filmmaker (director, editor, and actor), *m* is the *m* movies directed, written, and participated in by the filmmaker, *i* is the filmmaker’s serial number, *j* is the *j*-th movie, and *b_j_* is the *j*-th movie box office.
2. Production and distribution companies. The quality and publicity of the film depends on the production company and the distribution company. A good production company can guarantee the quality of movies, such as Huayi Brothers Media Group, Wanda Media Co. Ltd., August First Film Studio, etc. The greater the publicity of the movie by the distribution company, the more attention it will receive from the public, which will also have a certain impact on the box office.
3. Movie type. Everyone’s ability to appreciate movies is different. In the field of film and television, film and television appreciation are usually divided into cognitive appreciation, critical appreciation, and ablative appreciation. After investigation, the box office of science fiction, comedy, and action genres will be higher. The type of movie will also have a certain impact on the box office.

In addition, movie online word-of-mouth will also have an impact on movie box office. In the field of professional film and television appreciation, the plot and conflict of the film, the lens and the picture, the relationship between the characters in the film, the performance of the actors, the dubbing of the film and the music are more considered. Under normal circumstances, many ordinary viewers will post movie word-of-mouth reviews in online communities. The audience will comment using their viewing experience (Dewani et al., 2021). Most online film reviews contain spoilers. This spoiler information is information to resolve the uncertainty of the plot in advance. Ryoo et al. (2021) used the box office revenue and online word-of-mouth data of movies released in the United States to study the consequences of spoiler comments. In order to capture the degree of information in the spoiler review text to reduce the uncertainty of the drawing, a spoiler strength measurement is proposed. Related topic models are used and measured.

### Improved DSC-Transformer

**Figure 1:**
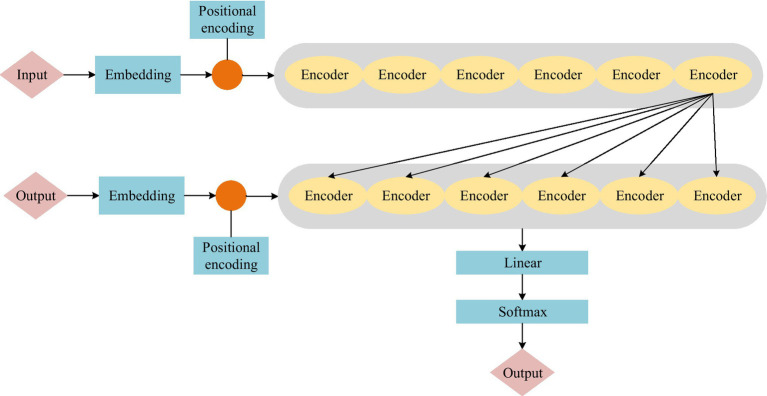
Transformer model structure diagram.

In 2017, Google proposed the Transformer model. This model can be used to extract features. It can solve the problem of slow training of recurrent neural network (RNN). The Transformer model structure is shown in Figure 1.

**Figure 2:**
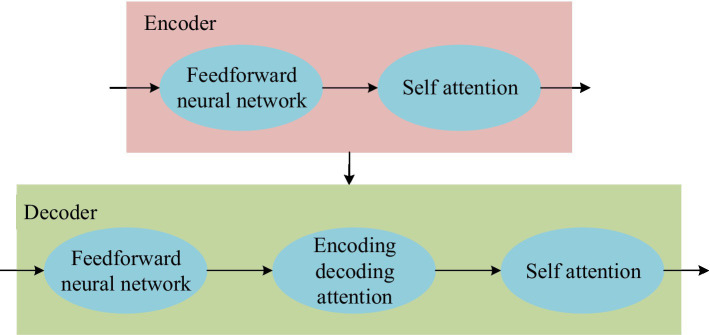
Partial structure of encoder and decoder.

The Transformer model structure is divided into two parts: encoding and decoding. Among them, the encoding part includes six encoders, and the decoding part also includes six decoders. Figure 2 shows the structure of the encoder and decoder.

**Figure 3:**
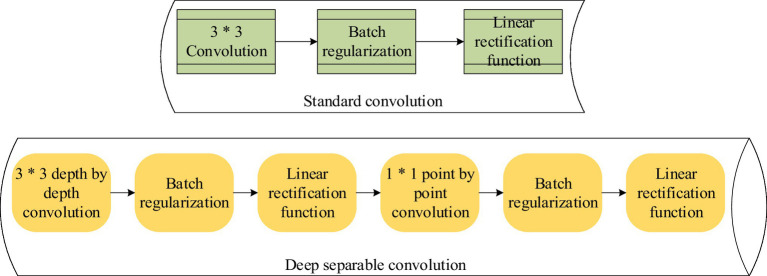
Process comparison between depthwise separable convolution (DSC) and standard convolution.

In Figure 2, the encoding part of the Transformer model uses a fully connected feedforward neural network. Fully-connected feedforward neural networks have problems such as many calculation parameters and difficulty in extracting local invariant features. Therefore, the model needs to be improved (Schwaller et al., 2019; Mousavi et al., 2020). DSC is used to replace the fully connected feedforward neural network. DSC is a model with few parameters and low computational cost for feature extraction. The model is composed of depthwise convolution (DW) and pointwise convolution (PW; Bai et al., 2018; Liu et al., 2019). The process comparison between DSC and standard convolution is shown in Figure 3.

Assume that the side length of the convolution kernel is D_K_. The number of input and output channels are M and N, respectively. The side length of the input feature map is D_F_. The calculation amount N_std_ of standard convolution can be expressed as Eq. (2):


(2) $$N_{std}=D_{K}\cdot D_{K}\cdot M\cdot D_{F}\cdot D_{F}$$

When using DSC calculation, filter the DW part, and perform channel conversion on the PW part. Their sizes are (D_K_, D_K_,1) and (1, 1, M) respectively. The numbers are M and N, respectively. These two quantities are calculated as Eqs. (3) and (4):


(3) $$N_{\mathrm{depthwise}}=D_{K}\cdot D_{K}\cdot M\cdot D_{F}\cdot D_{F}$$

(4) $$N_{point\mathrm{wise}}=M\cdot N\cdot D_{F}\cdot D_{F}$$

The total calculation amount can be expressed as Eq. (5):


(5) $$N_{ds}=N_{\mathrm{depthwise}}+N_{point\mathrm{wise}}$$

The ratio of the calculated amount of DSC and standard convolution is obtained, as in Eq. (6):


(6) $$\frac{N_{ds}}{N_{std}}=\frac{1}{N}+\frac{1}{D_{K}^{2}}$$

**Figure 4:**
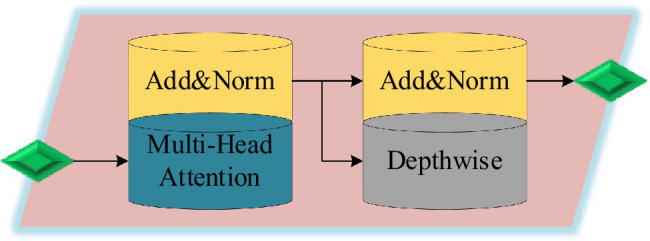
DSC-Transformer model.

DSC greatly reduces the amount of calculation and effectively reduces the consumption of computing resources. Therefore, using DSC to improve the Transformer model can achieve good calculation efficiency and improve accuracy (Kamal et al., 2019; Shang et al., 2020). The data are extracted with features, and then input into the DSC-Transformer model. The self-attention layer processes the input value to obtain a weighted feature vector. This vector and the original input form a skip connection structure. Then, the output of the previous layer is taken as the input into the DSC layer, and the final output of the DSC-Transformer model can be obtained after a series of summation and normalization processing. The structure of the DSC-Transformer model is shown in Figure 4.

### BERT Model

**Figure 5:**
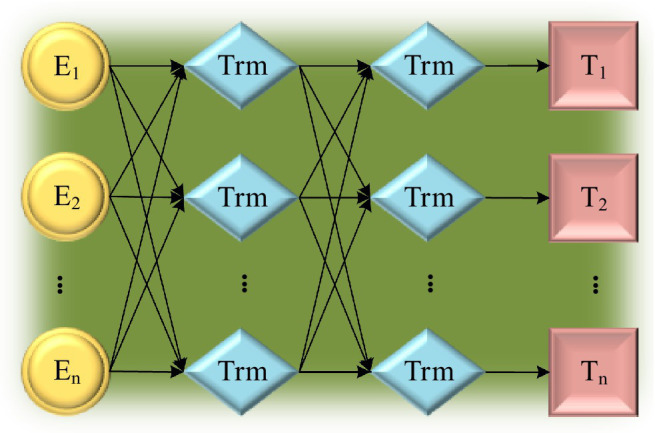
Bidirectional encoder representation from transformers (BERT) model.

BERT is a two-stage model. The first stage is to use the bidirectional language model for pre-training. The second stage uses a fine-tuning mode to solve specific downstream tasks (Cai et al., 2020; Mozafari et al., 2020; Liu et al., 2021; Yu et al., 2021). The BERT model uses a large amount of data for pre-training and combines the advantages of various models. Therefore, this model has achieved good results in many natural language processing tasks. The BERT model structure is shown in Figure 5. The first task is to randomly select some words to predict when inputting a sentence and then replace them with a special symbol. After that, the model learns the words that should be filled in these places according to the given labels. The second task adds a sentence-level continuity prediction task to the bidirectional language model, that is, predicting whether the two texts input to BERT is continuous texts. This task is introduced to allow the model to learn the relationship between consecutive text segments.

### Design of Box Office Prediction Model for Mixed-Feature Movies

The predictive features used include text features, continuous features, and discrete features. Therefore, these features need to be handled separately. Among them, the ideas of factorization machines (FM) and field-aware factorization machines (FFM) are used to process the discrete and continuous feature data. Firstly, the continuous and discrete features are, respectively, mapped to the first fully connected layer. The characteristics of the respective fields are constituted. These features are regarded as a learner and are all integrated into the second fully connected layer. A more powerful learner is formed.

For the text part, the BERT model is used to extract features. Two parts of the result ae obtained. Part of the result is the high-level text features of the serialized output. This part of the result is learned using a two-layer DSC-Transformer. The sequence feature of the text is obtained. However, another part of the result is the overall characteristics of the text obtained from the BERT model. Finally, the learning results of the two parts are spliced together and sent as input to the final fusion part. After two DSCs, the prediction result will be obtained. Continuous and discrete feature parts, text parts, and fusion parts are merged. The final mixed feature movie box office prediction model is composed.

### Data Acquisition and Processing

#### Feature Data Selection

Movie feature data are mainly from the piaofang.maoyan.com and movie.douban.com between 2017 and 2020. The selected feature information is shown in Table 1.

**Table 1:** The selected feature information.

	piaofang.maoyan.com page	movie.douban.com page
Feature information	Screening dates, working days and holidays, weekends, the number of days the movie has been released, the cumulative box office so far, the box office of the movie today, the proportion of the box office that day, average attendance, attendance, etc.	Movie name, introduction, director, starring role, genre, Douban rating, number of ratings, number of film reviews, number of short reviews, film length, release time, etc.

#### Data Acquisition

Selenium-based crawler technology is used to obtain data. Firstly, Chrome Driver is initialized. Then, find and locate elements step by step according to id, tagName, className, etc., until the obtained movie feature value data can be located. For some values that are too small, they can be directly removed, and the data that meet the conditions are stored. A total of more than 40,000 pieces of data have been obtained. These data are stored in Redis. List in Redis is used as a data structure, numbered as key. Each feature value is stored as a value, and these data are preprocessed and saved in csv format.

#### Loss Function

Usually, the loss function is used to evaluate the calculation method of the model. In deep learning, the loss function is used to quantify the difference in probability distribution between the predicted value of the model’s output and the actual label value. Different loss functions are applied in different occasions. The loss functions used are MSE and MAE. MSE measures the average of the square of the difference between the model predicted value and the actual value, while MAE is the average of the sum of the absolute values of the difference between the model predicted value and the actual value. Assuming real data $y=\left(y_{1},y_{2},\cdots ,y_{n}\right)$ , the fitted data are $\hat{y}=\left(y_{1},y_{2},\cdots ,y_{n}\right)$ . The calculation of MSE and MAE is shown in Eqs. (7) and (8):


(7) $$MSE=\frac{1}{n}\overset{n}{\underset{i=1}{\sum }}{\left({\hat{y}}_{i}-y_{i}\right)}^{2}$$

(8) $$MAE=\frac{1}{n}\overset{n}{\underset{i=1}{\sum }}\left|{\hat{y}}_{i}-y_{i}\right|$$

In general, the receptive field of the structure of one layer is limited, and it is difficult to integrate features of different depths. Multilayer structures are prone to overfitting. Therefore, the use of DW-Transformer models with different layers to learn high-order text features is compared, and the prediction results of the mixed-feature movie box office prediction model are compared. Since neural network models are often complex, computation requires a lot of computing resources and time. Therefore, the problem of efficiency has become an important problem that has plagued the field of deep learning for a long time. Hyperparameter tuning is very important. It can optimize the network model and improve the calculation speed and prediction accuracy. Additionally, the prediction accuracy of the model with different hyperparameters is tested. LSTM and Bi-LSTM are network structures that can process time-series data. Transformer models can be used to extract features. These three models are machine learning commonly used in movie box office predictions. Therefore, the established DSC-Transformer models are compared with them. In addition, the established model is compared with the prediction results of the machine learning method LightGBM. The established prediction model is used to predict the box office of some movies.

## Results and Discussion

### Comparison of DSC-Transformer Structure of Different Layers

**Figure 6:**
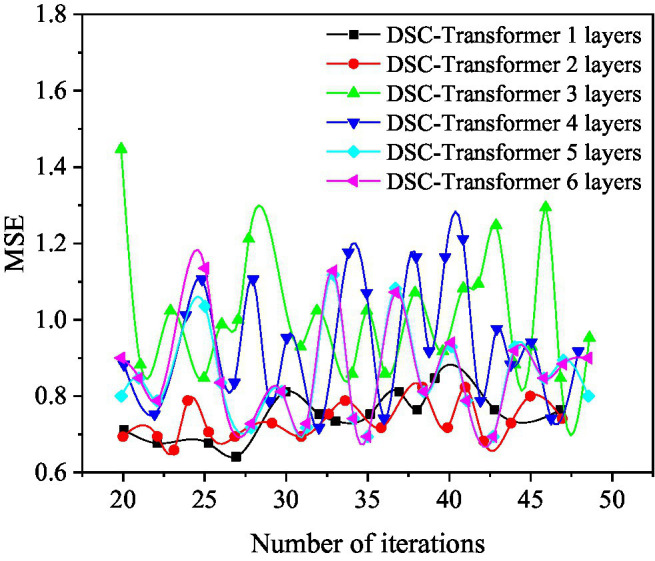
The mean square error (MSE) value of the DSC-Transformer structure with different layers used in the prediction model.

**Figure 7:**
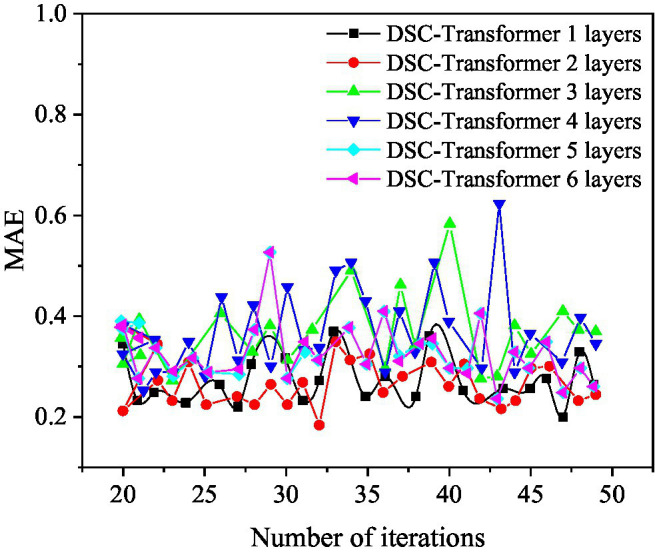
The mean absolute error (MAE) value of the DSC-Transformer structure with different layers used in the prediction model.

Different layers of DSC-Transformer models are used to learn high-level text features. In this case, the prediction results of the entire mixed feature movie box office prediction model are shown in Figures 6, 7.

Figure 6 shows that when a layer of DSC-Transformer is used to learn high-level text features, the corresponding MSE value is the best at 27 iterations, and its value is 0.6878. When using a two-layer DSC-Transformer to learn high-level text features, the corresponding MSE value is the best at 23 iterations, and its value is 0.6549. When using a three-layer DSC-Transformer to learn high-level text features, the corresponding MSE value is the best at 25 iterations, and its value is 0.7281. When using four-layer DSC-Transformer to learn high-level text features, the corresponding MSE value is the best when iterates 32 times, and its value is 0.6848. When using a five-layer DSC-Transformer to learn high-level text features, the corresponding MSE value is the best at 31 iterations, and its value is 0.6643. When the six-layer DSC-Transformer is used to learn high-level text features, the corresponding MSE value is the best at 35 iterations, and its value is 0.6941.

Figure 7 shows that when a one-layer DSC-Transformer is used to learn high-level text features, the corresponding MAE value is the best at 47 iterations, and its value is 0.1877. When using a two-layer DSC-Transformer to learn high-level text features, the corresponding MAE value is the best at 32 iterations, and its value is 0.1705. When using the three-layer DSC-Transformer to learn high-level text features, the corresponding MAE value is the best at 42 iterations, and its value is 0.2671. When using a four-layer DSC-Transformer to learn high-level text features, the corresponding MAE value is the best at 21 iterations, and its value is 0.2381. When using a five-layer DSC-Transformer to learn high-level text features, the corresponding MAE value is the best at 44 iterations, and its value is 0.2316. When the six-layer DSC-Transformer is used to learn high-level text features, the corresponding MAE value is the best at 43 iterations, and its value is 0.2362. When using the two-layer DSC-Transformer to learn high-level text features, the MSE and MAE values are both optimal, so the two-layer DSC-Transformer is the optimal design of the model.

### Hyperparameter Related Experiments

**Figure 8:**
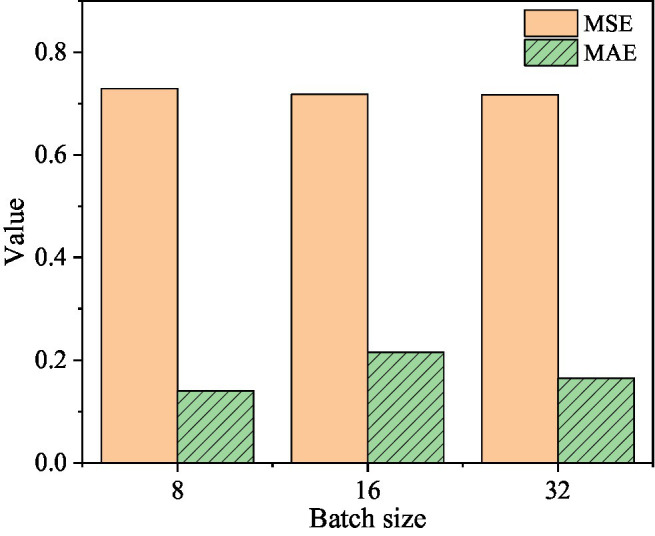
The corresponding MSE and MAE values when the learning rate is 1e-3.

**Figure 9:**
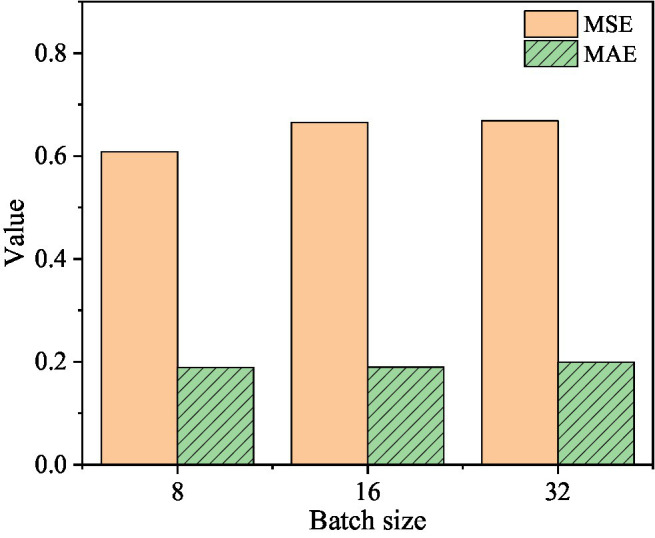
When the learning rate is 1e-4, the corresponding MSE and MAE values.

**Figure 10:**
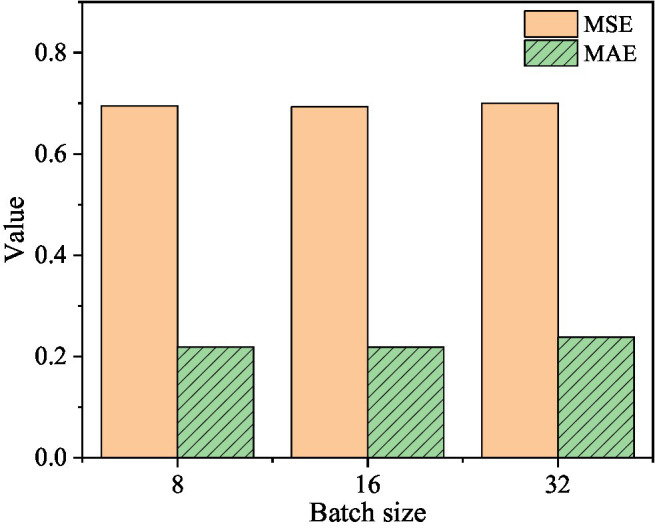
The corresponding MSE and MAE values when the learning rate is 1e-5.

In order to study the influence of the two parameters of learning rate and batch size on the prediction results of the two-layer DSC-Transformer structure learning high-level text feature model, the learning rate is selected as 1e-3, 1e-4, and 1e-5, and the batch size is 8, 16, and 32 samples for experiment. The specific results are shown in Figures 8–10.

Figure 8 shows that when the learning rate is 1e-3 and the batch size is 8, the corresponding MSE value is 0.729 and the MAE value is 0.14. When the batch size is 16, the corresponding MSE value is 0.7176 and the MAE value is 0.2152. When the batch size is 32, the corresponding MSE value is 0.7168 and the MAE value is 0.1646.

Figure 9 shows that when the learning rate is 1e-4 and the batch size is 8, the corresponding MSE value is 0.6081 and the MAE value is 0.1889. When the batch size is 16, the corresponding MSE value is 0.665 and the MAE value is 0.1896. When the batch size is 32, the corresponding MSE value is 0.6682 and the MAE value is 0.1993.

Figure 10 shows that when the learning rate is 1e-5 and the batch size is 8, the MSE value is 0.6945 and the MAE value is 0.219. When the batch size is 16, the corresponding MSE value is 0.693 and the MAE value is 0.2185. When the batch size is 32, the corresponding MSE value is 0.6998, and the MAE value is 0.2379. It can be concluded that: when the learning rate is 1e-4 and the batch size is 8, the MSE value of the mixed-feature movie box office prediction model is optimal; when the learning rate is 1e-3 and the batch size is 8, the box office of the mixed-feature movie is 8 The MAE value of the prediction model is optimal.

### Comparison of Results Using Different Algorithms

**Figure 11:**
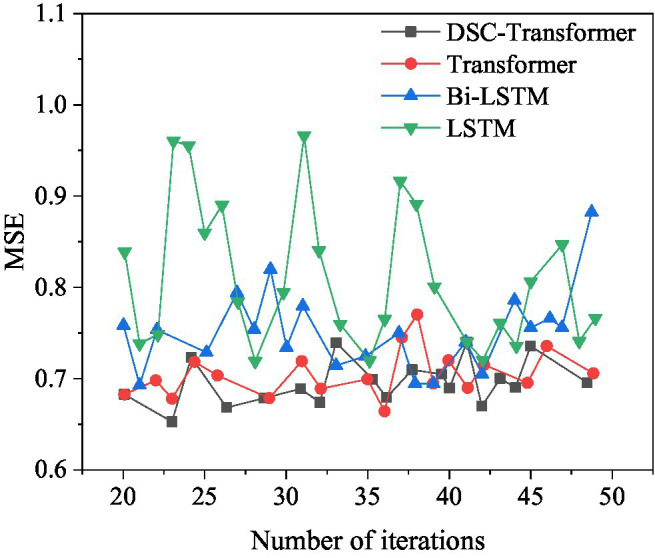
Comparison of MSE values corresponding to the model when using different algorithms.

**Figure 12:**
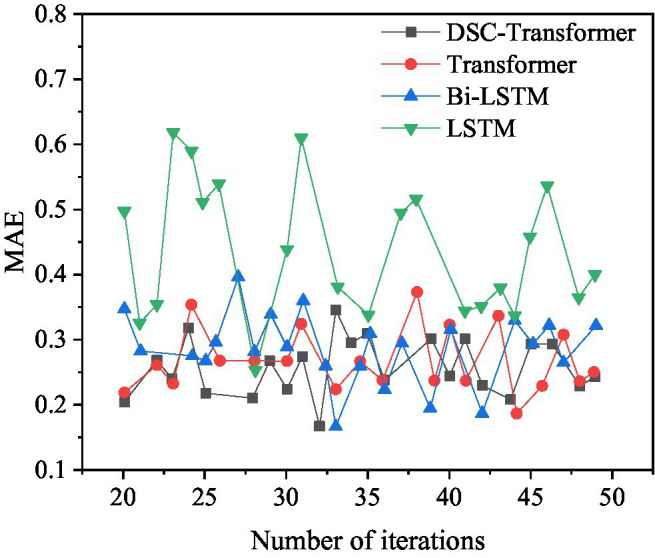
Comparison of MAE values corresponding to models when using different algorithms.

Different algorithm models are used to learn high-level text features, and the corresponding prediction results of the mixed feature movie box office prediction model are shown in Figures 11, 12.

Figure 11 shows that among the four different algorithm models, the optimal MSE value of the model using the DSC-Transformer is 0.6549. The optimal MSE value of the model used by Transformer is 0.6560. The optimal MSE value of the model using Bi-LSTM is 0.6843. The optimal MSE value of the model used by LSTM is 0.7064, and the MSE value of the model used by DSC-Transformer has achieved the best results. The prediction accuracy of the established DSC-Transformer model is higher than that of other algorithm models.

Figure 12 shows that among the four different algorithm models, the optimal MAE value of the model using the DSC-Transformer is 0.1706. The optimal MAE value of the model using Transformer is 0.1866. The optimal MAE value of the model using Bi-LSTM is 0.1758. The optimal MAE value of the model used by the LSTM is 0.3075, and the MAE value of the model used by the DSC-Transformer has achieved the best results. This shows that the prediction accuracy of the mixed feature movie box office prediction model using the DSC-Transformer model is higher than that of other algorithm models.

**Figure 13:**
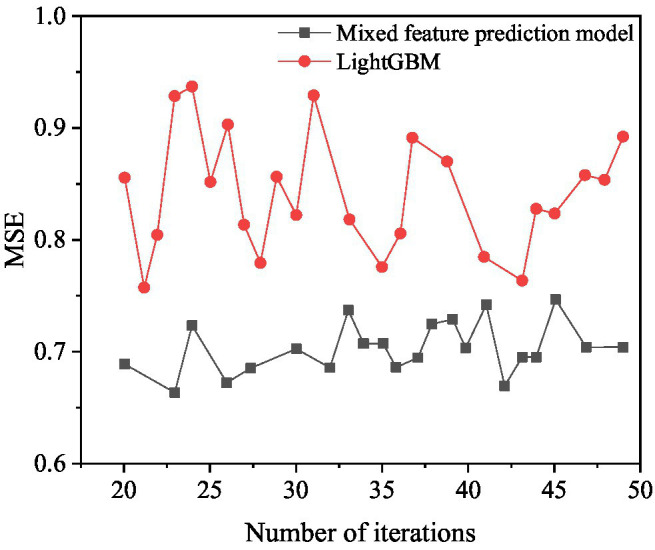
MSE results compared with LightGBM.

**Figure 14:**
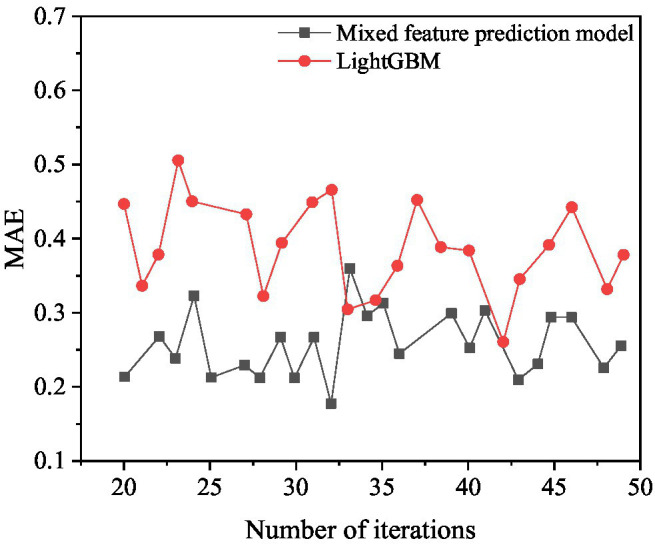
MAE results compared with LightGBM.

The box office prediction model of mixed features and LightGBM are used to predict the box office. The comparison results are shown in Figures 13, 14.

Figure 13 shows that when the number of iterations of the mixed feature movie box office prediction model is 23, the MSE value is optimal, and its value is 0.6625. When the number of iterations of LightGBM is 44, the MSE value is optimal, and its value is 0.7489. The mixed-feature movie box office prediction model is 13.04% higher than the prediction result of LightGBM.

Figure 14 shows that when the number of iterations of the mixed feature movie box office prediction model is 32, the MAE value is optimal, which is 0.1798. When the number of iterations of LightGBM is 43, the MAE value is optimal, and its value is 0.2581. The movie box office prediction model is 43.55% higher than the prediction result of LightGBM. In summary, the mixed feature movie box office prediction model proposed has higher prediction accuracy than LightGBM and can achieve good prediction results.

### Box Office Forecast

**Figure 15:**
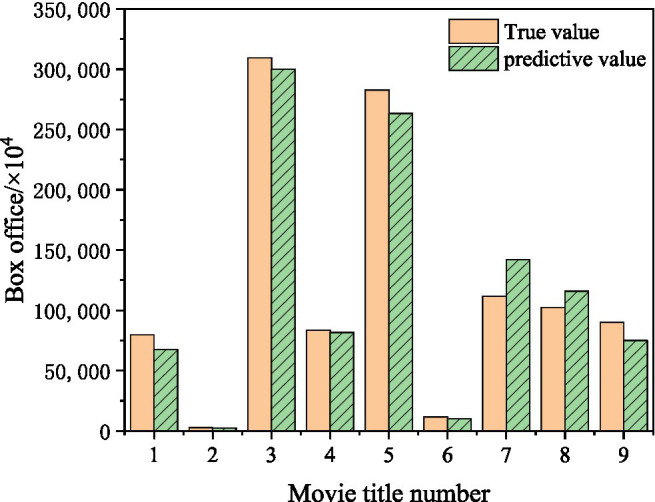
Movie box office forecast results.

In 2019, action and crime movies *P Storm* released, drama, suspense, crime movies *Hunt Down*, in 2020, war movies *the Eight Hundred* released, sports movies *Leap*, patriotic movies *My People My Homeland*, romance movie *the Story of Xi Bao*, war movie *the Sacrifice*, action, crime movie *Shock Wave 2*, drama genre *A Little Red Flower.* These nine movies are numbered 1–9, respectively. The proposed mixed-feature movie box office prediction model is used to predict the box office of these nine movies. The results are shown in Figure 15.

Figure 15 shows that the error between the predicted value and the true value of *4P Storm* is 15.3%. The error between the predicted value and the true value of *Hunt Down* is 10.5%. The error between the predicted value and the true value of *the Eight Hundred* is 3.1%. The error between the predicted value and the true value of *Leap* is 2.2%. The error between the predicted value and the true value of *My People My Homeland* is 6.8%. The error between the predicted value and the true value of *the Story of Xi Bao* is 9.8%. The error between the predicted value and the true value of *the Sacrifice* is 27.2%. The error between the predicted value and the true value of *Shock Wave 2* is 13.2%. The error between the predicted value and the true value of *A Little Red Flower* is 16.7%. Among them, *Leap* has the best prediction effect. *The Sacrifice* had the worst prediction effect. The error between the predicted value and the true value of other films is about 10%, which shows that the movie box office prediction model established has a good predictive effect.

## Conclusion

In recent years, the film industry has developed rapidly. The film production process itself is complex, involves many links, and takes a long time, making the film industry increasingly risky. Based on social network big data, it is established to analyze the movie box office and film and television review topics. Firstly, the factors that affect the movie box office are analyzed. Secondly, continuous and discrete feature parts, text parts, and fusion parts are merged. A mixed-feature movie box office prediction model based on deep learning is established to predict movie box office. Finally, the mixed feature movie box office prediction model based on deep learning is verified compared with other algorithms and models. When the learning rate is 1e-4, and the batch size is 8, the MSE value of the mixed-feature movie box office prediction model is optimal. The experimental results of hyperparameter correlation show that when the learning rate is 1e-3 and the batch size is 8, the MAE value of the mixed feature movie box office prediction model reaches the optimum. Compared with different algorithms, the prediction accuracy of the proposed mixed feature movie box office prediction model is higher than that of other algorithms, and it can achieve a good prediction effect. The proposed model can predict the box office of movies, thereby reducing investment risks. It is of great significance to film investors and social economy. There are still some shortcomings, such as marketing costs, production costs and other data are more difficult to obtain. Therefore, it is not very comprehensive when choosing the factors affecting the box office of a movie. In future research, some new influencing factors need to be added.

## Data Availability Statement

The original contributions presented in the study are included in the article/supplementary material, and further inquiries can be directed to the corresponding author.

## Author Contributions

All authors listed have made a substantial, direct, and intellectual contribution to the work, and approved it for publication.

## Funding

This work was supported by the National Social Science Fund of China of the Youth Project A Study of the Overseas Communication of Chinese Film and Television to Shape National Image (no. 20cxw010) and the National Ethnic Affairs Commission of the People’s Republic of China Project Political Security Governance in Minority Areas from the Perspective of Overall Security (no. 2021-GMB-009).

## Conflict of Interest

The authors declare that the research was conducted in the absence of any commercial or financial relationships that could be construed as a potential conflict of interest.

## Publisher’s Note

All claims expressed in this article are solely those of the authors and do not necessarily represent those of their affiliated organizations, or those of the publisher, the editors and the reviewers. Any product that may be evaluated in this article, or claim that may be made by its manufacturer, is not guaranteed or endorsed by the publisher.
